# α_1_-Microglobulin (A1M) Protects Human Proximal Tubule Epithelial Cells from Heme-Induced Damage In Vitro

**DOI:** 10.3390/ijms21165825

**Published:** 2020-08-13

**Authors:** Amanda Kristiansson, Sara Davidsson, Maria E. Johansson, Sarah Piel, Eskil Elmér, Magnus J. Hansson, Bo Åkerström, Magnus Gram

**Affiliations:** 1 Section for Infection Medicine, Department of Clinical Sciences, Lund University, 221 84 Lund, Sweden; sara.davidsson.913@gmail.com (S.D.); emjohansson22@gmail.com (M.E.J.); bo.akerstrom@med.lu.se (B.Å.); 2 Division of Hematology and Transfusion Medicine, Department of Laboratory Medicine, Lund University, 221 84 Lund, Sweden; 3 Mitochondrial Medicine, Department of Clinical Sciences Lund, Lund University, 221 84 Lund, Sweden; piels@email.chop.edu (S.P.); eskil.elmer@med.lu.se (E.E.); magnus.hansson@neurovive.com (M.J.H.); 4 Department of Anesthesiology and Critical Care Medicine, Center for Mitochondrial and Epigenomic Medicine, The Children’s Hospital of Philadelphia, Philadelphia, PA 19104, USA; 5 Department of Clinical Sciences Lund, Pediatrics, Lund University, 221 84 Lund, Sweden; magnus.gram@med.lu.se

**Keywords:** α_1_-microglobulin, oxidative stress, human kidney epithelial cells, antioxidant defense, heme, mitochondrial respiration

## Abstract

Oxidative stress is associated with many renal disorders, both acute and chronic, and has also been described to contribute to the disease progression. Therefore, oxidative stress is a potential therapeutic target. The human antioxidant α_1_-microglobulin (A1M) is a plasma and tissue protein with heme-binding, radical-scavenging and reductase activities. A1M can be internalized by cells, localized to the mitochondria and protect mitochondrial function. Due to its small size, A1M is filtered from the blood into the glomeruli, and taken up by the renal tubular epithelial cells. A1M has previously been described to reduce renal damage in animal models of preeclampsia, radiotherapy and rhabdomyolysis, and is proposed as a pharmacological agent for the treatment of kidney damage. In this paper, we examined the in vitro protective effects of recombinant human A1M (rA1M) in human proximal tubule epithelial cells. Moreover, rA1M was found to protect against heme-induced cell-death both in primary cells (RPTEC) and in a cell-line (HK-2). Expression of stress-related genes was upregulated in both cell cultures in response to heme exposure, as measured by qPCR and confirmed with in situ hybridization in HK-2 cells, whereas co-treatment with rA1M counteracted the upregulation. Mitochondrial respiration, analyzed with the Seahorse extracellular flux analyzer, was compromised following exposure to heme, but preserved by co-treatment with rA1M. Finally, heme addition to RPTE cells induced an upregulation of the endogenous cellular expression of A1M, via activation of the nuclear factor erythroid 2-related factor 2 (Nrf2)-pathway. Overall, data suggest that A1M/rA1M protects against stress-induced damage to tubule epithelial cells that, at least partly, can be attributed to maintaining mitochondrial function.

## 1. Introduction

Oxidative stress is a term often used to describe physiological conditions where there is an imbalance between oxidants and antioxidants that results in a biological stress response with disturbed redox signaling, and downstream molecular damage [1]. The production of oxidants, including reactive oxygen species (ROS) and free radicals, can be induced by external sources, such as radiation or smoke, as well as a wide variety of internal biological processes, such as inflammation, and may have detrimental effects on cells and tissues when not balanced by the antioxidation defense systems [2].

Both acute kidney injury (AKI) and chronic kidney disease (CKD) have been associated with decreased antioxidant defense and/or increased ROS that contribute to the disease progression [3,4]. AKI is defined as a sudden loss of renal function and CKD as decreased function, i.e., glomerular filtration rate (GFR) <60 mL/min/1.73 m^2^, or presence of kidney damage markers for more than 3 months [5,6]. Occurrence of AKI has also been shown to increase the risk of developing CKD, exacerbating pre-existing CKD, and accelerating the progression to end-stage renal disease (ESRD) [7,8,9].

The etiologies of AKI are multifactorial and diverse, for example after serious illness, e.g., sepsis, ischemic injury in connection with major surgery or due to nephrotoxic drugs [10]. Additionally, the release of excessive amounts of free heme from myoglobin can result in rhabdomyolysis-induced AKI [11]. Furthermore, studies on patients that develop AKI after cardiopulmonary bypass surgery have shown increased levels of cell-free hemoglobin and markers of oxidative stress in the plasma of these patients [12]. Cell-free hemoglobin and free heme constitute potent endogenous generators of oxidative stress, due to the highly redox active iron-ion [13]. Thus, generation of ROS by released heme-proteins is considered an important feature of AKI.

Despite the heterogeneity of AKI, oxidative stress and mitochondrial dysfunction are considered hallmarks of AKI pathophysiology. Since the kidney is a highly metabolic organ, it is rich in mitochondria [14,15]. The proximal tubules generate a large amount of ATP through oxidative phosphorylation, to support active transport within the respiratory chain, and are therefore a major site of ROS production [16]. ROS constitute a central signaling mechanism in cells, however when excessive ROS are produced, they may become toxic to both the mitochondria and the cell [15]. Under physiologic conditions, ROS activate transcription of defense mechanisms-related genes, e.g., superoxide dismutase and glutathione, via nuclear factor erythroid 2-related factor 2 (Nrf2) [15,17]. However, excessive ROS generation may exhaust these protective mechanisms, leading to damage of the respiratory chain and thus a further aggravation of the oxidative stress.

The antioxidant α_1_-microglobulin (A1M) is a radical scavenger and heme-binder [18,19]. A1M is a 26 kDa protein that is mainly synthesized in the liver, and is part of the lipocalin protein family [20]. Following synthesis, A1M is released into the blood where it is found in complexes with IgA, prothrombin or albumin, or remains unbound in micromolar concentrations (1–2 μM) [21,22,23]. A1M is rapidly equilibrated between the intra- and extravascular compartments and is found in most cells and tissues of the body, including skin, kidneys and placenta [24,25]. A1M is filtered in the glomeruli, and its main catabolic route is reabsorption by the proximal tubular cells and degradation. However, a small percentage is excreted with the urine, and therefore A1M can be used as a marker of tubular damage [26].

A1M has been shown to protect cell cultures in vitro, including K562 cells, erythrocytes and keratinocytes, against oxidative and heme-induced damage [27,28,29]. In addition, reparative/regenerative effects have been described for A1M. These effects are believed to be dependent on its reducing activity, and a number of physiological substrates have been described, including oxidized forms of the heme proteins cytochrome *c* and methemoglobin, free iron and oxidized collagen [28,30]. A1M can be internalized by cells, and bind to a subunit of Complex I in the mitochondria, where it has been shown to protect mitochondrial function [31]. In animal studies, intravenously administered recombinant human A1M (rA1M) has been shown to be distributed to the kidneys, and confer protection against radiation-, preeclampsia- and rhabdomyolysis-induced kidney injuries [32,33,34]. These studies suggest that the protection by rA1M is due to its heme- and radical-binding properties, its reductase activity and its localization to and protection of mitochondria. It has also been shown that infused rA1M is found in renal tubular epithelial cells within a few minutes [35]. In this study, we have further characterized the renal protective mechanisms of A1M/rA1M by exposing human proximal tubule epithelial cells, both a cell line and primary cells, to heme-induced oxidative stress.

## 2. Results

### 2.1. rA1M Confers Cytoprotection of HK-2 Cells after Exposure to Heme

**Figure 1:**
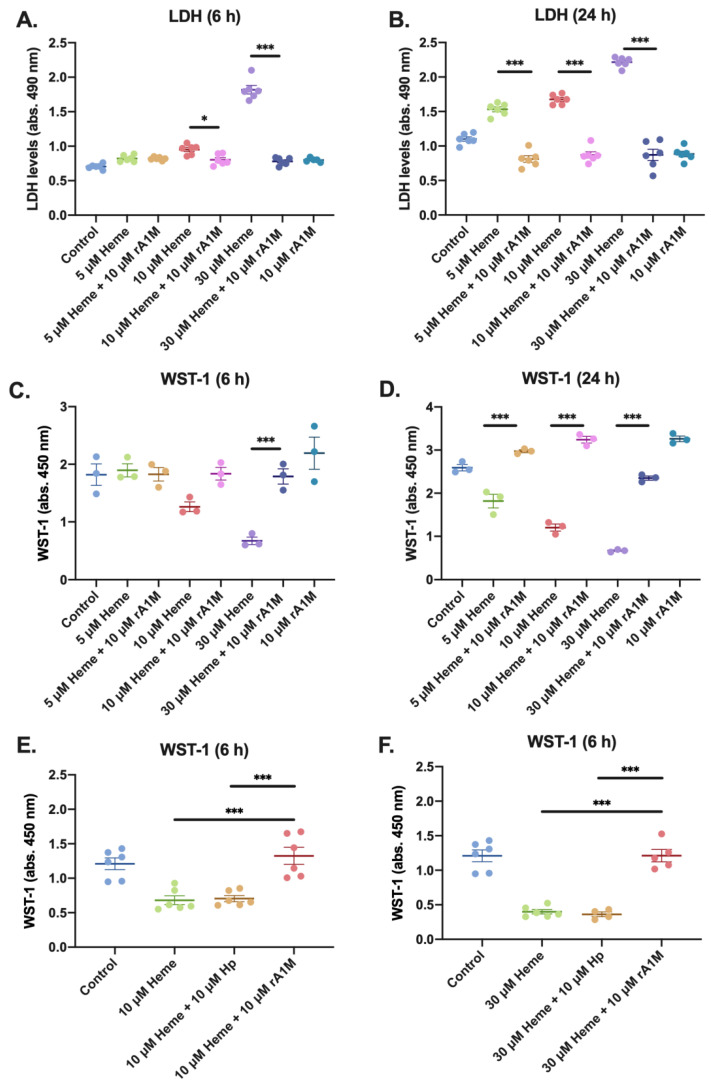
Cytoprotective effects of rA1M after exposure to heme. HK-2 cells were exposed to 5, 10 or 30 μM heme with or without simultaneous addition of 10 μM rA1M (**A**–**F**) or 10 μM Hp (**E**–**F**) and cell death was analyzed by LDH levels (**A**–**B**) in cell culture media, and cell viability was measured by WST-1 (**C**–**F**) after 6 h (**A**,**C**,**E**,**F**) and 24 h (**B**,**D**). Values are presented as mean ± SEM, LDH *n* = 6, WST *n* = 3–6. Differences between groups were analyzed using one-way ANOVA with post hoc Sidak. * *p* < 0.05, *** *p* < 0.001.

To examine the cytoprotective effects of rA1M, HK-2 cells were exposed to 5, 10 or 30 μM heme, with or without the simultaneous addition of 10 μM rA1M. The rA1M concentration was selected to correspond to concentrations reported in in vivo studies [32,33,36]. Cell death was measured by lactate dehydrogenase (LDH) leakage into the cell culture media. In line with previous studies [27,28], heme induced a dose-dependent increase in LDH levels after 6 and 24 h exposure (Figure 1A,B). This was, however, significantly counteracted at both time-points, following co-treatment with rA1M. Analysis of cell viability using the water-soluble tetrazolium salt (WST-1) assay displayed similar results as LDH (Figure 1C,D), i.e., a decreased cell viability at 6 h following exposure to 30 μM heme and with all concentrations at 24 h. Co-treatment with rA1M restored cell viability to control levels at both time-points, and at all heme concentrations. Exposure of cells to rA1M (10 μM) only showed no difference as compared to control cells in this or any of the following experiments. To investigate if the protective effects of rA1M was specific or caused by a “general protein buffering protection”, the plasma protein haptoglobin (Hp) was used as a comparison. Contrary to rA1M, Hp had no cell protective effect against 10 or 30 μM heme-induced reduction in cell viability, as measured by WST-1 (Figure 1E,F).

### 2.2. rA1M Counteracts Heme-Induced Upregulation of HSP70 and HMOX-1 Transcripts

**Figure 2:**
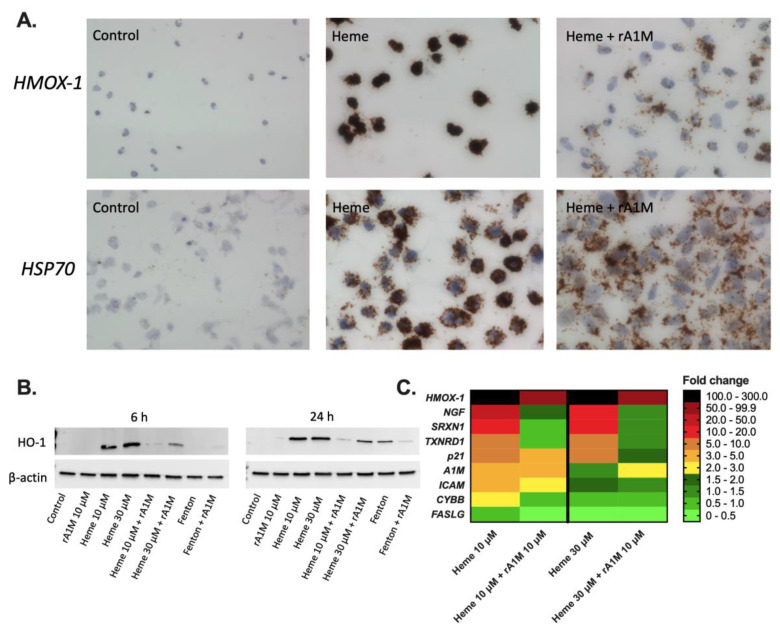
rA1M counteracts induction of stress and apoptosis in HK-2 cells. Representative light microscopy images (magnification 20X) of in situ visualization (**A**) of *HSP70* and *HMOX-1* RNA levels in HK-2 cells exposed to culture medium only (Control), 10 μM heme (Heme) or 10 μM heme with 10 μM rA1M (Heme + rA1M). Western blot (**B**) of HO-1 from HK-2 total cell extracts following exposure to 10 μM heme or Fenton reaction-induced hydroxyl radicals (200 μM ammonium iron(III) sulfate dodecahydrate, 400 μM hydrogen peroxide, and 2 mM ascorbic acid) for 6 and 24 h, with or without simultaneous addition of 10 μM rA1M. Notably, β-actin was used as protein load control. Membranes are from a representative analysis and β-actin was analyzed on the same experimental samples. Densitometric quantifications and uncropped membranes are presented in Supplementary Figures S1 and S2. Heat map (**C**) of mRNA expression of the genes (*HMOX-1, NGF, SRXN1*, TXNRD1, *p21, A1M*, *ICAM-1*, *CYBB* and *FASLG)* of HK-2 cells exposed to 10 μM heme, with or without 10 μM rA1M for 4–6 h (*n* = 2). Data were normalized to *GAPDH* and presented as fold change 0–300, as indicated by colors, compared to control cells.

RNAscope, an in situ hybridization assay for the detection of RNA levels within intact cells, was used to evaluate expression of heat shock protein 70 (*HSP70*) and heme oxygenase 1 (*HMOX-1*) in HK-2 cells, following exposure to 10 μM heme, with or without the simultaneous addition of 10 μM rA1M for 2 h (Figure 2A). Staining of *HSP70* and *HMOX-1*-mRNA, biomarkers of heme- and oxidative stress, was found to be increased in cells exposed to heme, as compared to controls, i.e., cells without heme addition, indicating upregulation of these genes. Co-treatment with rA1M reversed this effect, suggesting that rA1M counteracts the heme-induced upregulation of *HSP70* and *HMOX-1*.

Protein analysis of HO-1, using Western blot, showed a congruent increase of protein levels after exposure to 10 and 30 μM heme, at both 6 and 24 h, compared to control cells (Figure 2B). The band intensity was reduced following the simultaneous addition of 10 μM rA1M at both time-points, indicating that rA1M inhibits the heme-induced increase of HO-1 protein levels. In addition to heme, oxidative stress induced by Fenton reaction-generated hydroxyl radicals were studied. The addition of the Fenton reactants (200 μM ammonium iron(III) sulfate dodecahydrate, 400 μM hydrogen peroxide, and 2 mM ascorbic acid) caused an increase of HO-1 protein levels at 24 h, but not at 6 h, as compared to the control cells. Co-treatment with 10 μM rA1M suppressed the Fenton-induced increase of HO-1 protein levels at 24 h.

In addition to in situ hybridization, expression of stress response genes in HK-2 cells was also analyzed by qPCR (Figure 2C) after 4–6 h exposure to 10 and 30 μM heme, with or without simultaneous addition of 10 μM rA1M. In line with RNAscope, upregulation of *HMOX-1* was seen after exposure to heme. This upregulation was partially counteracted with the addition of rA1M. Sulfiredoxin 1 (*SRXN1*), thioredoxin reductase 1 (*TXNRD1*) and nerve growth factor (*NGF*) displayed upregulated expression after exposure to heme, which was reduced down to the level of controls by rA1M. The expression of cyclin-dependent kinase inhibitor 1 (*p21*) was also upregulated following exposure to heme, with reduced expression in the presence of rA1M. Fas ligand (*FASLG*) showed no clear increase in expression in response to heme-induced stress. The A1M gene (*A1M*) showed an increased expression in the heme-exposed HK-2 cells. However, the expression was not decreased with rA1M addition. Intercellular adhesion molecule 1 (*ICAM-1*) was increased in the HK-2 cells in response to heme addition and rA1M inhibited the increase. Cytochrome b-245 beta (*CYBB*) expression, finally, was slightly increased following exposure to 10 μM heme and in samples where rA1M were present; the expression was normalized.

### 2.3. rA1M Protects Mitochondrial Function during Heme Exposure of HK-2 Cells

Oxygen consumption rates (OCR) were determined following exposure to heme at different concentrations for 6 or 24 h, with or without rA1M co-treatment. A respirometric protocol was used to derive various parameters of mitochondrial respiration with the XF96 Seahorse analyzer, as described in the methods section. Following measurements with the Flux analyzer, total protein content of the wells was determined. In line with the viability assays, a lower protein content in wells with cells exposed to heme without rA1M was observed (see Supplementary Figure S3). Hence, the flux analysis data are presented as OCR per μg/mL protein.

**Figure 3:**
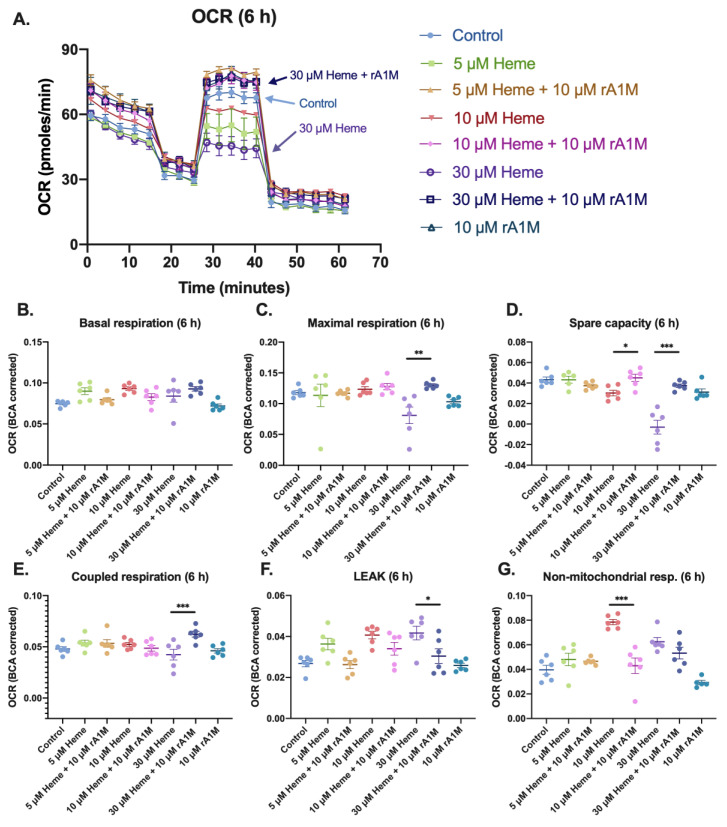
rA1M protects mitochondrial function after heme addition. Mitochondrial respiration (**A**) after incubation with heme (5, 10 or 30 μM), with or without rA1M (10 μM) after 6 h. Data are corrected for protein content (BCA), as described in the methods section, and presented as basal respiration (**B**), maximal respiration (**C**), coupled respiration (**D**), spare capacity (**E**), proton leak (**F**) and non-mitochondrial respiration (**G**). Values are from a representative experiment and presented as mean ± SEM (*n* = 6, analyzed on the same plate). Differences between groups were analyzed using one-way ANOVA with post hoc Sidak (**B**–**F**), * *p* < 0.05, ** *p* < 0.01, *** *p* < 0.001.

The OCR, a measurement of the function of the respiratory chain, is shown in Figure 3A. The OCR was reduced with increasing concentrations of heme (5–30 μM), and preserved at control levels when 10 μM rA1M was added to cells for 6 h (Figure 3A). OCR measurements from each well were calculated and corrected for protein content. Subsequently, the OCR linked to phosphorylation activity of the ATP-synthase (coupled respiration) and the spare respiratory capacity was calculated (Figure 3). At 6 h, basal respiration was not significantly affected by heme (Figure 3B), while the maximal respiration decreased following exposure to the highest concentration of heme (30 μM) and was preserved with the addition of rA1M (Figure 3C). The spare capacity was clearly reduced upon the addition of heme (10 and 30 μM) (Figure 3D) and preserved with co-treatment with rA1M. OCR linked to coupled respiration was also significantly lower in cells incubated with 30 μM heme compared to 30 μM heme + rA1M (Figure 3E), although, it seems from the graph that the main effect is caused by the increase in the rA1M group. Heme additionally caused a significantly increased LEAK, which was kept at control levels with simultaneous administration of rA1M (Figure 3F). Non-mitochondrial respiration (NMR) was increased with 10 and 30 μM heme, as compared to control cells and co-treatment with rA1M, significantly reduced the effects of 10 μM heme, but not 30 μM (Figure 3G).

### 2.4. rA1M Reduces Heme-Induced Cell Death in Human Primary Renal Proximal Tubule Epithelial Cells

**Figure 4:**
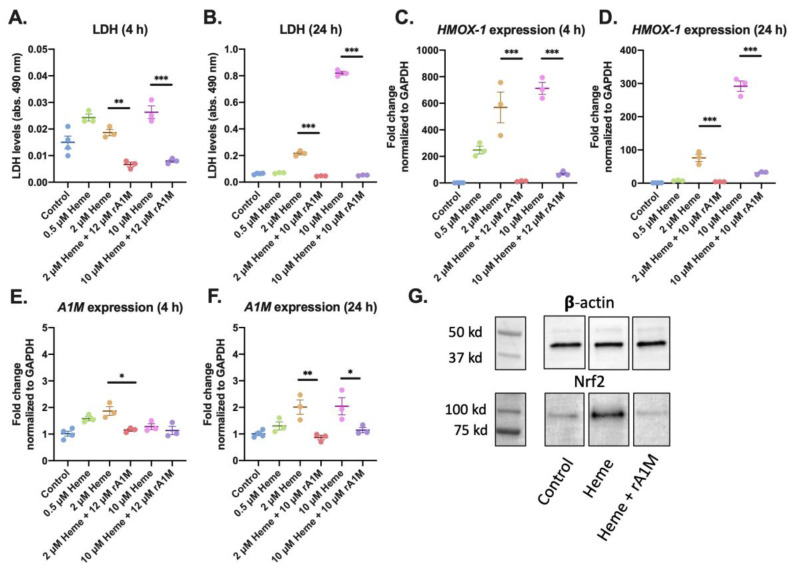
rA1M protects human primary RPTE cells following exposure to heme. RPTEC were incubated with increasing concentrations of heme (0.5–10 μM), with or without addition of rA1M (10 or 12 μM). Cell viability was analyzed by LDH release into the culture media after 4 h (**A**) and 24 h (**B**). Gene expression of *HMOX-1* (**C**–**D**) and *A1M* (**E**–**F**) was measured by qPCR, after 4 h (**C**,**E**) and 24 h (**D**,**F**). Protein levels of Nrf2 was analyzed by Western blot (**G**) from RPTE cell lysates following exposure to 2 μM heme, with or without co-treatment with 10 μM rA1M. Moreover, β-actin was used as a protein loading control. All samples were applied to one membrane. Membranes are from a representative analysis and β-actin was analyzed on the same experimental samples. Densitometric quantification and uncropped membrane are presented in Supplementary Figure S4. Values are presented as mean ± SEM (*n* = 3, analyzed on the same plate). Differences between groups were analyzed using one-way ANOVA with post hoc Sidak (**A**–**F**), * *p* < 0.05, ** *p* < 0.01, *** *p* < 0.001.

To further evaluate the biological relevance of our findings in primary cells, studies using human primary renal proximal tubule epithelial cells (RPTEC) were performed. Cells were exposed to heme-induced oxidative stress with or without co-treatment with rA1M and subsequently analyzed with regards to cell death (LDH release) and stress response (qPCR analysis of *HMOX-1* and *A1M*). These cells were more sensitive to heme than HK-2 cells, and the range 0.5–10 μM heme was therefore selected for the further analysis. An increase in LDH release was observed after exposure to 2 and 10 μM of heme at both 4 and 24 h (Figure 4A,B). Co-treatment with 12 μM rA1M significantly prevented the LDH release. Furthermore, the *HMOX-1* mRNA expression increased in a dose-dependent manner following exposure to heme (0.5–10 μM), that could be significantly inhibited by simultaneous addition of 10–12 μM rA1M (Figure 4C,D). In line with previous studies [37], *A1M* expression also increased at both 4 and 24 h after exposure to increasing amounts of heme (0.5–10 μM), and was counteracted with addition of 10–12 μM rA1M (Figure 4E,F).

The Nrf2 transcription factor regulates the expression of antioxidant proteins in response to inflammation and injury caused by oxidative damage [38]. Measurement of Nrf2 protein levels was performed by Western blot, following heme exposure and incubation for 4 h, with or without rA1M, (Figure 4G). An intense Nrf2 band was seen after exposure to 2 μM heme, which was reduced in intensity to similar levels as in control cells after co-treatment with 10 μM rA1M.

Previous studies have shown that oxidatively challenged cells internalize A1M [27]. Radioimmunoassay (RIA) was used to determine if rA1M was taken up by RPTE cells after 2 h of incubation with 2 μM heme and 10 μM rA1M. RIA data was normalized to protein content, measured by BCA protein assay, and showed that cells internalized A1M (2.54 ng A1M/μg protein), see also Supplementary Figure S5. The low content of A1M in control cells may correspond to endogenous A1M in the cells.

## 3. Discussion

Oxidative stress is a mechanism inherent to many disorders of cellular injury, and considered pathophysiologically important in the development of many renal diseases [39,40]. Therefore, it is of interest to find new treatment strategies targeting oxidative stress. Notably, rA1M, a recombinant form of the human heme-binding and radical scavenging protein A1M, has shown promising preclinical results in reducing kidney damage [32,33,34]. In this work, we have characterized the protective effects of rA1M in vitro, and show that it counteracts heme-induced cell death, mitochondrial deficiency and cellular stress in proximal tubule epithelial cells, both HK-2 cells and a primary human cell culture.

Free heme can be cytotoxic via the formation of ROS. Although not specifically investigated here, this probably involves the formation of superoxide or singlet oxygen. It addition, heme has been described to generate hydroxyl radicals, via the Fenton reaction [41,42]. Moreover, due to its lipophilic structure, heme can intercalate cell membranes, resulting in lipid and protein peroxidation or DNA damage (reviewed in [43]). In line with previous descriptions [44,45], we observed a heme-induced concentration-dependent cell-death of proximal tubule epithelial cell cultures that could be counteracted by co-treatment with rA1M at all heme concentrations tested. In order to characterize the rA1M protective effects further, the response of a number of heme-induced stress- and injury related markers was evaluated.

Analysis showed that the mRNA expression of stress response genes was upregulated in response to heme exposure. These included *HMOX-1*, an intracellular heme detoxification protein [11,12,27,46,47]; *SRXN1* and *TXNRD1*, part of the antioxidant defense regulated by Nrf2 [48]; *NGF*, involved in cell proliferation, survival and inflammatory response [49] and involved in apoptotic response [50]; *p21*, a cellular stress response gene and cell cycle regulator; *CYBB*, a catalytic membrane-bound subunit of NADPH oxidase (Nox2) involved in the electron transport across biological membranes generating superoxide [51]; and *ICAM-1*, an endothelial- and leukocyte-associated transmembrane protein involved in the immune response and cell migration. Upregulation of *HMOX-1* and *HSP70*, well-known markers of cellular stress [52,53], was also confirmed within intact cells using an in situ mRNA expression analysis. Co-treatment with rA1M inhibited or reduced the increased expression of all markers. Increased HO-1 was also seen at the protein level in the HK-2 cells. Interestingly, an increased expression of the *A1M* gene in response to heme stress was also observed. Previous studies have reported that ROS and heme induce upregulation of the *A1M* gene in other cells, and it has been suggested that this is a part of the cellular response in the defense against free heme [28,37]. Co-treatment with rA1M normalized the *A1M* expression in RPTE cells, but not in HK-2 cells. Taken together, these data suggest a heme-induced cellular stress that is normalized or reduced with co-treatment of rA1M.

Nrf2 has been proposed to mediate protection against heme- and hemoglobin-induced ROS, by promoting *HMOX-1* and *A1M* gene expression [54,55]. During normal conditions, Kelch-like ECH-associated protein 1 (KEAP1) dimers facilitate ubiquitinoylation of Nrf2 and subsequent degradation. However, with increasing oxidative stress, KEAP1 is covalently modified and unable to ubiquitinylate Nrf2, leading to the accumulation of intracellular Nrf2 and translocation to the nucleus [54]. This was also indicated in our data; Nrf2 protein was more abundant in RPTE cells exposed to heme than control cells. Co-treatment with rA1M normalized Nrf2 levels, suggesting a reduction in cellular oxidative stress. Nrf2 has also been described to have a role in maintaining mitochondrial homeostasis and structural integrity (reviewed in [56]), highlighting the connection between oxidative stress and the mitochondria.

The role of mitochondrial dysfunction in the pathology of AKI is well-established. AKI has been associated with a destruction of mitochondrial structure, imbalance in mitochondrial dynamics (fission and fusion), impairment of mitochondrial ATP production, reduction of mitochondrial biogenesis, release of cytochrome *c* and increased mitochondrial ROS production [57,58,59,60]. Preserving mitochondrial function has been reported by others to translate to the improvement of renal function, reduction of inflammation and fibrosis, decrease in renal injury markers and increased survival in animal models of sepsis-, ischemia reperfusion injury-, and drug-induced AKI [57]. In line with the study by Olsson et al. [31], this study demonstrates a protective effect of rA1M against heme-induced mitochondrial dysfunction. More specifically, we show that, during heme challenge, rA1M appears to preserve the maximal respiration and spare capacity, as well as to prevent an increase in LEAK respiration, an indicator of the integrity of the inner mitochondrial membrane. We also observed that rA1M normalized the NMR. This latter may be explained by the reduced induction of HO-1. In the degradation of heme, HO-1 consumes three molecules of oxygen per molecule of heme [53]. Our data showed a dose-dependent *HMOX-1* gene-expression increase in response to heme, similar to the increase in NMR from 5 to 10 µM, but not 30 µM, heme. If the reduction in NMR after addition of 30 µM heme reflects decreased *HMOX-1* gene expression, the depletion of HO-1 and other detoxification proteins, or a change in cell viability cannot be conclusively concluded here. A limitation of the flux analysis experiments in the current study is that we did not specifically evaluate if the mitochondrial protective effects were rA1M specific by comparing with control proteins. To elucidate if the mitochondrial protection is provided by the heme scavenging or antioxidative properties of rA1M, further studies are needed. However, based on the study by Olsson et al. [31] and the cell protective effects observed here, as compared to Hp (see Figure 1), it can be reasoned that the mitochondrial protection is rA1M specific and not a “protein buffering effect”.

Several potential mechanisms for the protective effects of rA1M against the heme-induced oxidative damage in the tubular cell cultures can be considered. Based on previous knowledge of the antioxidation mechanisms of the protein [61], extracellular scavenging of heme-groups, ROS and other downstream oxidants, the binding of cytosolic heme-groups and oxidants, reduction/repair/regeneration of cellular oxidative lesions, or direct interaction with mitochondrial components, all could contribute to the protection. Here, we observed a complete inhibition of cell death using three-fold less rA1M compared to heme. Considering available data on heme-binding [19], it is therefore not likely that extracellular scavenging of heme groups is the only protective mechanism employed by rA1M. It has previously been shown that exogenously added rA1M is taken up and internalized by blood cells [29,31,37], and the results presented here show that the RPTE cells internalize A1M in the presence of heme. Hence, it is possible that internalized, cytosolic rA1M participates in reducing oxidants and oxidation lesions in the cytosol, as part of its protective properties. In addition, it has been shown that internalized rA1M is localized to mitochondria, and specifically bound to complex I (NADH dehydrogenase) of the respiratory chain, where it preserves mitochondrial function during heme- and ROS-induced oxidative stress [31]. In this study, we observed that rA1M prevented heme-induced impairment of mitochondrial respiration. Mitochondrial complex I is known as a major site of mitochondrial-related ROS [62]. Therefore, it is intriguing to speculate that the specific binding of rA1M to mitochondrial complex I protects this complex from oxidative injury and thus, prevents further oxidative injury resulting from heme-induced, complex I-related mitochondrial dysfunction.

In conclusion, heme-mediated oxidative stress results in cell lysis, disrupted mitochondrial respiration and induction of cellular stress response in proximal tubule epithelial cells, which were all reduced or prevented by co-treatment with a recombinant form of the human antioxidant A1M. Increasing evidence suggests that combating the imbalance between oxidants and antioxidants in kidney diseases could improve outcome for patients. Therefore, together with recent in vivo studies [32,33,34], the current study supports the use of rA1M in the treatment of renal diseases.

## 4. Materials and Methods

### 4.1. A1M

Recombinant human A1M (rA1M) with an N-terminal His-tag [63] was expressed, purified, and refolded from *Escherichia coli* cultures, as described by Åkerström et al. [33]. The endotoxin content was determined using the Limulus Amebocyte Lysate test kit (LAL QCL-1000, Lonza, Basel Switzerland) according to the manufacturer’s description, and was below 0.1 EU/mg protein. The final protein solutions were sterile filtered through a 0.22 μm filter (Merck Millipore, Darmstadt, Germany).

### 4.2. Materials

Hydrogen peroxide (30%) and L-Ascorbic acid were from Sigma Aldrich (St-Louis, MO, USA). Ammonium iron(III) sulfate dodecahydrate was from Merck Millipore. Heme (Ferriprotoporphyrin IX chloride) was purchased from Porphyrin Products, Inc (Logan, UT, USA) and prepared by dissolving in dimethyl sulfoxide (Sigma-Aldrich). Haptoglobin (Hp) was from pooled human plasma (Sigma-Aldrich).

### 4.3. Cell Cultures

Human kidney cortex proximal tubule epithelial cells (HK-2, ATCC CRL-2190, ATCC, United Kingdom) were cultured according to the instructions from the manufacturer in keratinocyte serum-free medium, with added bovine pituitary extract (0.05 mg/mL) and epidermal growth factor (5 ng/mL) (all from Invitrogen, United Kingdom). Human primary renal proximal tubule epithelial cells (RPTEC, ATCC PCS-400-010, ATCC) were cultured according to the instructions from the manufacturer. RPTE cells were grown in renal epithelial cell basal media, supplemented with renal epithelial cells growth kit components and antimicrobials: gentamicin (10 μg/mL), amphotericin B (0.275 μg/mL), penicillin (10 units/mL) and streptomycin (10 μg/mL) (all from ATCC). Cell cultures were incubated at 37 °C in the presence of 5% CO_2_. When cells reached 80–90% confluence, heme (0.5–30 μM, from a freshly prepared 10 mM stock solution), or a mixture of ammonium iron(III) sulfate dodecahydrate, hydrogen peroxide, and ascorbic acid (generating hydroxyl radicals via the Fenton reaction), with or without addition of either Hp (10 μM) or rA1M (10 or 12 μM) or rA1M only, was added, and cells were incubated for up to 24 h. For details on exposure concentrations and time, see the respective figure.

### 4.4. RNA Isolation and Quantitative PCR (qPCR)

Cell cultures were harvested using QIAzol-lysis reagent for RNA extraction (QIAGEN, Germantown, MD, USA), following the manufacturer’s instructions. Total RNA was isolated from HK-2 and RPTE cells, using Directzol RNA MiniPrep (Zymo Research, Irvine, CA, USA), followed by RNeasy Mini Kit (QIAGEN). Furthermore, cDNA was synthesized from 1 μg total RNA using the iScript cDNA Synthesis Kit (Bio-Rad, Hercules, CA, USA Rad). RT2 qPCR Primer Assay primers from QIAGEN were used to quantify the mRNA expression of *HMOX-1* (or HO-1 for protein), *A1M/AMBP*, *CYBB*, *FASLG*, *SRXN1* and TXNRD1. Primers from Eurofins MWG Operon were used to quantify the mRNA expression of *ICAM-1*, *p21* and *NGF*. Data were normalized to glyceraldehyde-3-phosphate dehydrogenase (*GAPDH*, RT2 qPCR Primer Assay from QIAGEN). Expression was analyzed using iTaq Universal SYBR Green Supermix (Bio-Rad). Amplification was performed as described by the manufacturer (Bio-Rad) for 40 cycles in an iCycler Thermal Cycler (Bio-Rad) and data analyzed using iCycler iQ Optical System Software. Data are presented as fold change values (2ˆ(–ΔΔCT) method) compared to control cells.

### 4.5. RNAscope

Cells were seeded on chamber slides (Fisher Scientific, Hampton, New Hampshire, USA) (25,000 cells/well). Samples were incubated with heme (10 μM), with or without rA1M (10 μM) for 2 h. After incubation, probes were added (positive, negative, *HMOX-1* and *HSP70*) and procedure was performed according to the manufacturer’s instructions for the RNAscope**^®^** 2.0 Assay (Advanced Cell Diagnostics Inc, Hayward, CA, USA).

### 4.6. Cell Viability Assays

Cell proliferation reagent WST-1 (Roche Diagnostics GmbH, Mannheim, Germany) was used according to the instructions from the manufacturer. LDH release was measured using the CytoTox 96-Non-Radio Cytotoxicity Assay (Promega Biotech AB, Nacka, Sweden), according to the manufacturer’s instructions. Absorbance was measured at 450 nm for WST-1 and 490 nm for LDH (VICTOR 1420 multilabel plate reader, PerkinElmer Life Sciences, Waltham, MA, USA).

### 4.7. Flux Analysis

**Figure 5:**
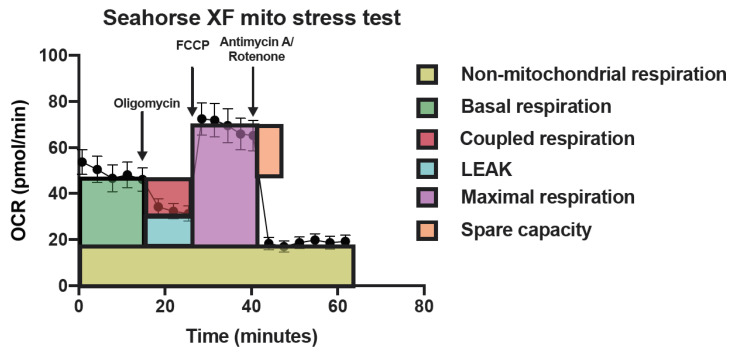
Graphical overview of measurements from the flux analysis. Graphical description of how calculations of the following mitochondrial parameters were made: non-mitochondrial respiration (values after antimycin A), basal respiration (baseline-antimycin A), coupled respiration (baseline-oligomycin), LEAK (oligomycin-antimycin A), maximal respiration (FCCP-antimycin A) and spare capacity (FCCP-baseline). One timepoint before and one timepoint after each injection were chosen for calculations. Calculations were normalized to protein content in the wells after measurements with the Flux analyzer.

A XF96 extracellular flux analyzer (Agilent Technologies, Santa Clara, CA, USA) was used to measure the cellular oxygen consumption rate (OCR). When cells reached 80–90% confluency, HK-2 cells were seeded out in a XF96 plate at 25,000 cells per well in culture medium without supplements the day before the experiment and incubated at 37 °C and 5% CO_2_ overnight. Heme (5–30 µM), with or without the addition of rA1M (10 µM) or rA1M only, was added to the cells (medium only was added to control cells) for 6 or 24 h, after which the medium was exchanged for XF-Base medium (Agilent Technologies, DMEM), containing 10 mM glucose, 2 mM L-glutamine and 5 mM pyruvate, set to pH 7.4. Following measurements of basal respiration, the respiration, which is dependent on the endogenous substrate supply at resting state, the ATP-synthase inhibitor oligomycin (1 µg/mL), the protonophore fluoro-carbonyl cyanide phenylhydrazone (FCCP) (0.125 µM), the complex I inhibitor rotenone (2 μM) and the complex III inhibitor antimycin A (1 µg/mL) were added sequentially to assess basal routine respiration, LEAK respiration (the oxygen consumption related to the flux of protons through the mitochondrial membrane that is not linked to proton shuttling at the ATP-synthase), electron transport system capacity (maximal respiration) and NMR, respectively. Subsequently, coupled respiration, the respiration linked to phosphorylation activity by the ATP synthase [64], as well as spare respiratory capacity, defined as the difference in respiration between basal and maximal respiration [65], were calculated. All data was corrected for NMR and an overview of calculations are presented in Figure 5. After measurements with the Flux analyzer, total protein content of the wells was quantified as described below.

### 4.8. Total Protein Extraction and Analysis

Cells were washed three times with phosphate buffered saline (PBS) and lysed on ice (60 min) in a cell extraction buffer (Invitrogen), containing a protease inhibitor cocktail (Roche), and then centrifuged (13,000 rpm, 10 min, 4 °C). Protein concentrations were quantified in the supernatants with a BCA protein assay kit, according to instructions from the manufacturer (Thermo Scientific, Waltham, MA, USA). Absorbance was measured at 550 nm (VICTOR 1420 multilabel plate reader, PerkinElmer Life Sciences).

### 4.9. SDS-PAGE and Western Blot

SDS-PAGE was performed as previously described with pre-cast stain-free 4–20% gels and with β-mercaptoethanol in the sample buffer and heat denaturated samples (Bio-Rad) and ran in duplicates [66]. Precision Plus Protein All Blue Prestained Protein Standards were used for the size determination of proteins (Bio-Rad). After transfer to polyvinylidene difluoride (PVDF) membranes by electroblotting (Transblot^®^ Turbo, Bio-Rad), membranes were incubated in blocking solution (5% non-fat dry milk (Bio-Rad) in PBS-T), followed by a monoclonal rabbit anti-human Nrf2, (1:1000; GenWay, San Diego, CA, USA), mouse anti-human HO-1 (1:1000; Abcam, Cambridge, UK) or mouse anti-human β-actin (1:1000; Abcam) antibody. Swine anti-rabbit IgG-HRP (Dako, Glostrup, Denmark), goat anti-mouse IgG-HRP (Dako) or goat anti-mouse IgG-Alexa Fluor 647 (Life Technologies, Carlsbad, CA, USA) were used as secondary antibody. Signals from HRP-conjugates were detected using Clarity Western ECL Substrate (Bio-Rad). Membranes and gels were imaged and analyzed using the ChemiDoc™ MP System (Bio-Rad).

### 4.10. Radioimmunoassay (RIA)

The A1M concentration of RPTEC protein extracts was determined using RIA, as described previously [67]. Briefly, human urine A1M (purified as previously described) [68] was labelled with ^125^I, according to the chloramine T-method [69]. Separation of iodine-labelled proteins from free iodide was achieved by gel chromatography on a Sephadex G-25 column (PD-10, GE Healthcare, Chicago, IL, USA). Standard A1M concentrations or unknown samples were mixed with goat anti-A1M-serum, diluted 1:6000 in RIA buffer (0.1 M phosphate, pH 7.4, 0.1% BSA, 0.02% NaN_3_), and ^125^I-labelled A1M (0.1–0.2 MBq/μg protein). Following incubation (overnight, room temperature), bovine serum and 15% polyethylene glycol in RIA buffer was added and the samples were centrifuged (2500× *g*, 40 min). The ^125^I activity was measured using a Wallac Wizard 1470 gamma counter (Perkin-Elmer Life Sciences).

### 4.11. Statistical Analysis

Data are presented as mean ± SEM. Statistical significance was calculated with a one-way ANOVA test corrected for multiple comparisons (Sidak), comparing samples with equal concentration of heme, with or without rA1M in addition to comparing to control. In figures, statistical significance compared to control is not shown. Statistical analysis was performed with GraphPad Prism (GraphPad Prism 8.3.0 for MacOS; GraphPad Software; GraphPad, Bethesda, MD, USA). Values of *p* < 0.05 were considered significant and are marked in the figures.

## Supplementary Materials

The following are available online at https://www.mdpi.com/1422-0067/21/16/5825/s1, Figure S1: Uncropped western blots and densitometric calculation from Figure 2 (6 h), Figure S2: Uncropped western blots and densitometric calculation from Figure 2 (24 h), Figure S3: BCA results used for flux analysis normalization, Figure S4: Uncropped western blots and densitometric calculation from figure 4, Figure S5: Uptake of A1M in RPTE cells analyzed with radioimmunoassay.
