# Supplementary material for: α1-Microglobulin (A1M) Protects Human Proximal Tubule Epithelial Cells from Heme-Induced Damage In Vitro
Source: Int J Mol Sci. 2020 Aug 13;21(16):5825. doi: 10.3390/ijms21165825 (PMC7461577; doi:10.3390/ijms21165825)
Supplement: Supplementary file 1 [file ijms-21-05825-s001.pdf]

Supplementary Material

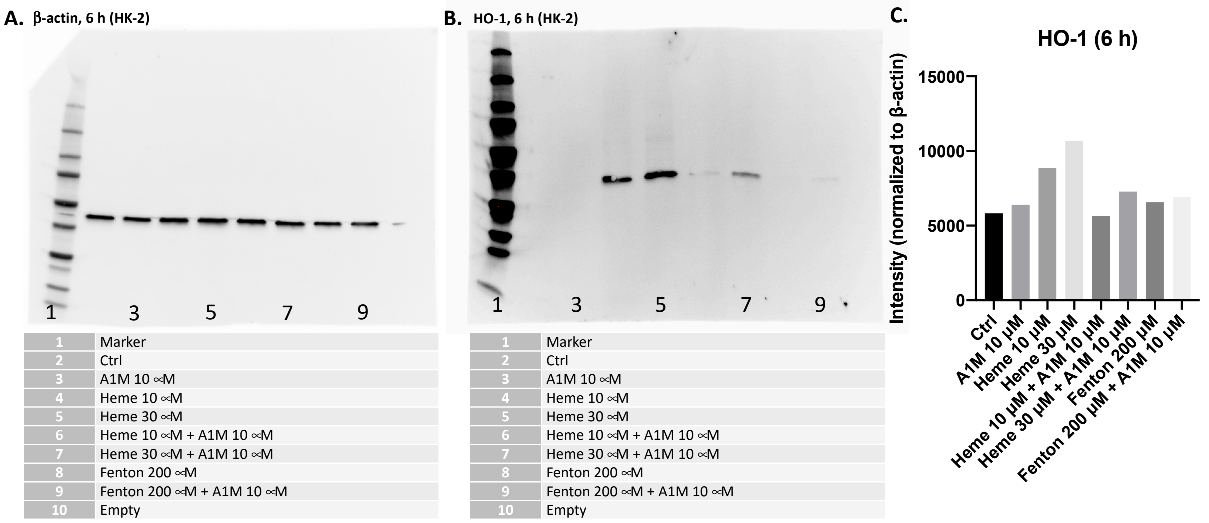

Supplementary figure S1. Uncropped western blots from figure 2 with β-actin (**A**) and HO-1 (**B**) after 6 h. The bar graph represents densitometric calculations of HO-1 normalized to β-actin (**C**) presented as intensity.

A. β-actin, 24 h (HK-2)

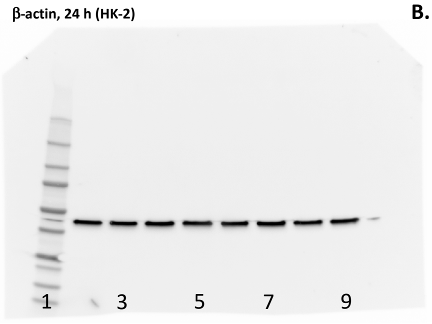

1	Marker
2	Ctrl
3	A1M 10 ∝M
4	Heme 10 ∝M
5	Heme 30 ∝M
6	Heme 10 ∝M + A1M 10 ∝M
7	Heme 30 ∝M + A1M 10 ∝M
8	Fenton 200 ∝M
9	Fenton 200 ∝M + A1M 10 ∝M
10	Empty

B. HO-1, 24 h (HK-2)

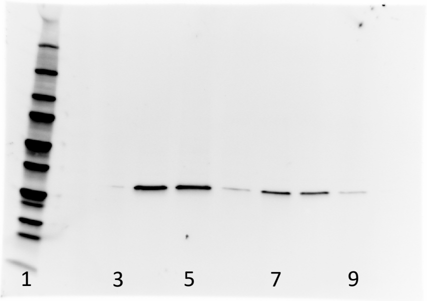

1	Marker
2	Ctrl
3	A1M 10 ∝M
4	Heme 10 ∝M
5	Heme 30 ∝M
6	Heme 10 ∝M + A1M 10 ∝M
7	Heme 30 ∝M + A1M 10 ∝M
8	Fenton 200 ∝M
9	Fenton 200 ∝M + A1M 10 ∝M
10	Empty

C.

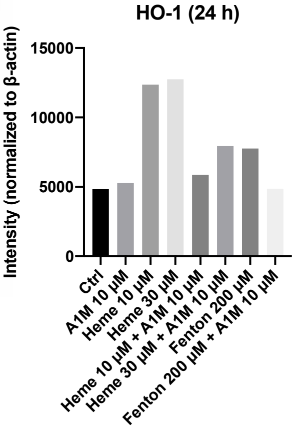

Supplementary figure S2. Uncropped western blots from figure 2 with β-actin (**A**) and HO-1 (**B**) after 24 h. The bar graph represents densitometric calculations of HO-1 normalized to β-actin (**C**) presented as intensity.

A.

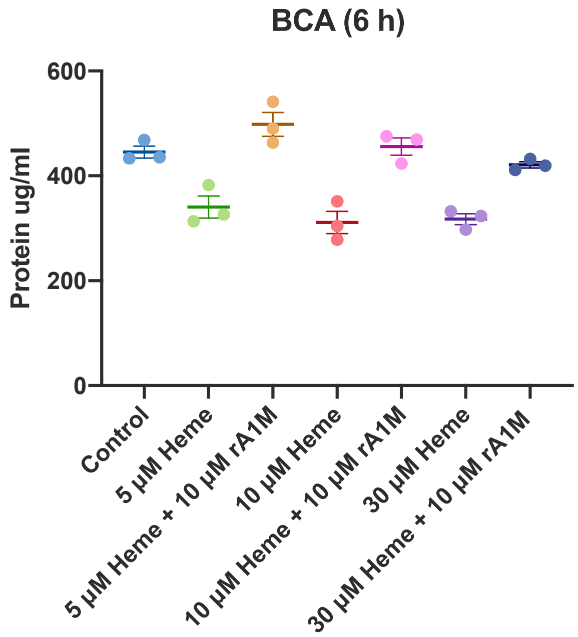

B.

Sample	µg/ml
Control	445
Heme 5 µM	340
Heme 10 µM	311
Heme 30 µM	317
Heme 5 µM + A1M	498
Heme 10 µM + A1M	455
Heme 30 µM + A1M	420
A1M	569

Supplementary figure S3. BCA results used for Seahorse normalization. BCA was measured after incubation with heme with or without A1M (**A**) and the calculated mean (**B**) was used to correct OCR after treatment and incubation (n = 3).

A. β-actin, 4 h (RPTEC)

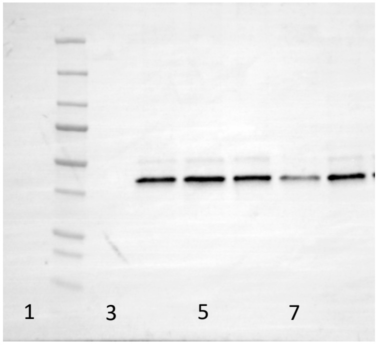

1	Empty
2	Marker
3	Empty
4	Control
5	10 ∝M A1M
6	2 ∝M Heme
7	Excluded – too low protein amount (30 ∝M heme)
8	2 ∝M Heme + 10 ∝M A1M

B. Nrf2, 4 h (RPTEC)

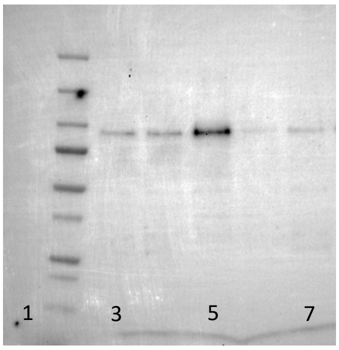

1	Empty
2	Marker
3	Control
4	10 ∝M A1M
5	2 ∝M Heme
6	Excluded – too low protein amount (30 ∝M heme)
7	2 ∝M Heme + 10 ∝M A1M

C.

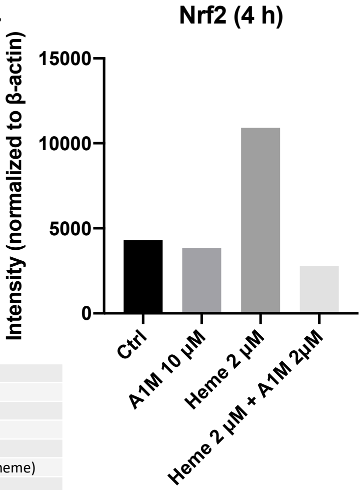

Supplementary figure S4. Uncropped western blots from figure 4 with β-actin (**A**) and Nrf2 (**B**) after 4 h. The bar graph represents densitometric calculations of Nrf2 normalized to β-actin (**C**) presented as intensity with control set to 1.

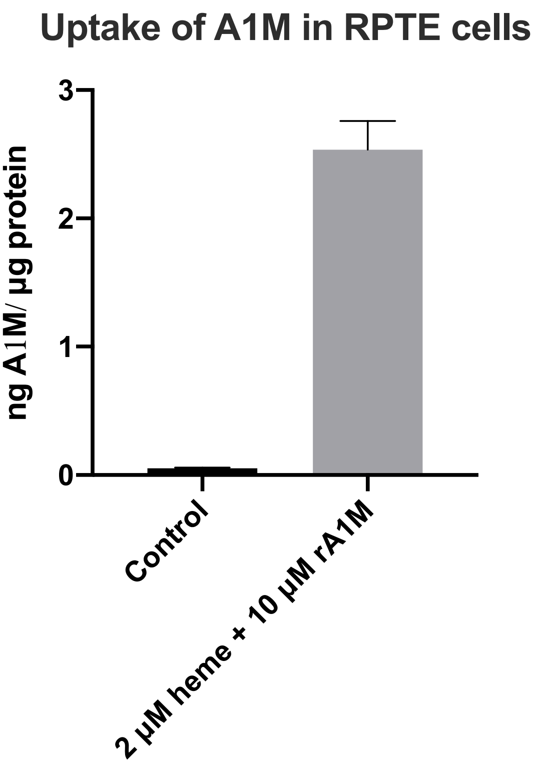

Supplementary figure S5. Uptake of A1M in RPTE cells analyzed with radioimmunoassay. Cells were incubated 2 h with 2 µM heme and 10 µM rA1M. Results are adjusted for BCA content and presented as mean (± SEM).
